# LONGITUDINAL CHANGES IN WELL-BEING OF PARENTS OF INDIVIDUALS WITH DEVELOPMENTAL OR MENTAL HEALTH PROBLEMS

**DOI:** 10.1093/geroni/igz038.1814

**Published:** 2019-11-08

**Authors:** Carlie J Sloan, Marsha R Mailick, Jinkuk Hong, Jung-Hwa Ha, Jan S Greenberg, David M Almeida

**Affiliations:** 1 The Pennsylvania State University, University Park, Pennsylvania, United States; 2 University of Wisconsin - Madison, Madison, Wisconsin, United States; 3 Seoul National University, Seoul, Korea, Republic of

## Abstract

The negative impact of having a child with special needs on parental well-being is well documented. Previous research has suggested age attenuation of these impacts. However, this has not yet been examined longitudinally in late life. Therefore, it is unclear how the effect of having a child with a developmental disability or mental health problem changes as parents age and children become less likely to live at home. Using responses from the Study of Midlife in the United States (MIDUS), this study investigates: (1) longitudinal changes in the effect of having a child with a developmental or mental health problem on parental well-being, (2) age and gender moderations on these effects, and (3) the unique impact of factors directly related to the child’s condition. Multiple linear regressions revealed that having a child with a developmental disability was predictive of higher negative affect, more somatic symptoms, and lower psychological well-being longitudinally. Additionally, there was a main effect of having a child with a mental health problem in predicting higher negative affect. However, age moderations were revealed such that the effect of having a child with a developmental disability or mental health problem was diminished for older parents. Additionally, within-group analyses revealed that longer duration of developmental disabilities and later parental age of onset of mental health problems were predictive of better outcomes. Overall, results suggest that although having a child with special needs is related to poorer well-being, these effects can attenuate as parents age and adapt.

